# A mixed-reality surgical trainer with comprehensive sensing for fetal laser minimally invasive surgery

**DOI:** 10.1007/s11548-018-1822-7

**Published:** 2018-07-27

**Authors:** Allan Javaux, David Bouget, Caspar Gruijthuijsen, Danail Stoyanov, Tom Vercauteren, Sebastien Ourselin, Jan Deprest, Kathleen Denis, Emmanuel Vander Poorten

**Affiliations:** 1 Department of Mechanical Engineering, KU Leuven, Leuven, Belgium; 2 Department of Circulation and Medical Imaging, Norwegian University of Science and Technology, Trondheim, Norway; 3 Wellcome / EPSRC Centre for Interventional and Surgical Sciences (WEISS), UCL, London, UK; 4 Department of Development and Regeneration, Woman and Child, Biomedical Sciences, KU Leuven, Leuven, Belgium

**Keywords:** Fetal minimally invasive surgery, Mixed-reality trainer, Face validation, Content validation

## Abstract

**Purpose:**

Smaller incisions and reduced surgical trauma made minimally invasive surgery (MIS) grow in popularity even though long training is required to master the instrument manipulation constraints. While numerous training systems have been developed in the past, very few of them tackled fetal surgery and more specifically the treatment of twin-twin transfusion syndrome (TTTS). To address this lack of training resources, this paper presents a novel mixed-reality surgical trainer equipped with comprehensive sensing for TTTS procedures. The proposed trainer combines the benefits of box trainer technology and virtual reality systems. Face and content validation studies are presented and a use-case highlights the benefits of having embedded sensors.

**Methods:**

Face and content validity of the developed setup was assessed by asking surgeons from the field of fetal MIS to accomplish specific tasks on the trainer. A small use-case investigates whether the trainer sensors are able to distinguish between an easy and difficult scenario.

**Results:**

The trainer was deemed sufficiently realistic and its proposed tasks relevant for practicing the required motor skills. The use-case demonstrated that the motion and force sensing capabilities of the trainer were able to analyze surgical skill.

**Conclusion:**

The developed trainer for fetal laser surgery was validated by surgeons from a specialized center in fetal medicine. Further similar investigations in other centers are of interest, as well as quality improvements which will allow to increase the difficulty of the trainer. The comprehensive sensing appeared to be capable of objectively assessing skill.

## Introduction

Found in monochorionic twins, TTTS causes an unbalanced blood flow due to undesired vascular anastomoses on the placenta. One of the fetuses, referred to as the *donor*, transfers his nutrition to the other fetus, the *recipient*. When untreated, perinatal death happens in 90% of the cases, while there is as much as 50% chance of neurological impairment in case of survival [18]. Minimally invasive fetal surgery leads to improved survival rate and does not increase the risk for preterm birth as much as open surgery would [1]. Nevertheless, as in all MIS procedures, instrument handling requires significant skill.

**Fig. 1:**
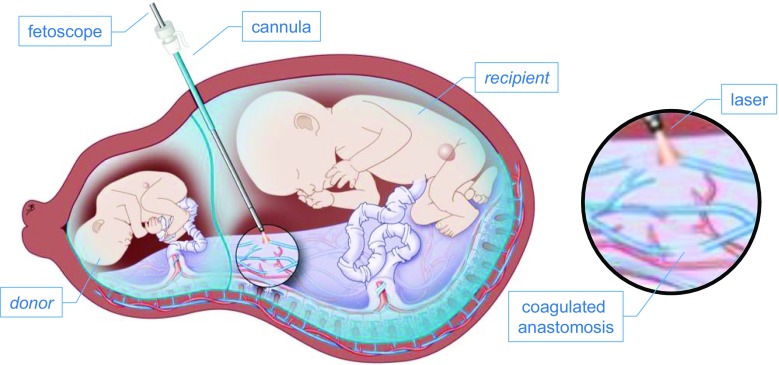
Two fetuses are represented in a womb. A flexible cannula is inserted through the maternal abdomen allowing surgeons to access the placenta with a fetoscope equipped with a scope and laser to coagulate specific targets (courtesy of UZ Leuven)

As illustrated in Fig. 1, the intervention is performed using a thin flexible cannula inserted through the maternal abdomen allowing the surgeon to access the *recipient’s* amniotic sac with a fetoscope. The latter is an endoscope used within a sheath protecting the scope but also providing an additional port [10] giving the possibility to equip fetoscopes with an instrument such as a therapeutic laser fiber. The surgeon then sweeps the placental surface by delicately manipulating the fetoscope and coagulates specific targets by actuating the therapeutic laser by pressing a foot pedal. To avoid preterm premature rupture of membranes (PPROM), one can only use small diameter scopes ($<3$ mm) [6], implicating a limited image quality. Because of poor illumination and the turbidity of the amniotic fluid through which one operates, the visualization during this procedure is notoriously bad, making the scope navigation more difficult. In addition, the thick maternal abdomen severely limits the workspace of the scope. Scopes are quite commonly damaged beyond repair when too large stresses are applied to view remote sites. Also, the risk for PPROM could rise as similar large stresses are applied on the fetal membranes.

Overall, the skill required for mastering TTTS treatment is quite significant, while the number of cases is quite low as 20% of spontaneous twin pregnancies are monochorionic and only 15% of these complicate into TTTS [4]. Moreover, an increased interest of surgeons willing to train for fetal laser surgery is anticipated due to an economic growth in various countries and the increasing knowledge on this procedure [16]. As such, the availability of training systems dedicated to such highly specific surgeries appears to be mandatory for reaching a sufficient level of proficiency, hence ensuring a better patient outcome as long as fewer surgical adverse events. In fact, the traditional surgical training model by demonstration “see one, do one, teach one” [11] would imply too long periods of training and thus preferably be replaced by a model referred to as “Practice makes perfect” [23], placing the practice on trainers at a higher level of importance.

For classic MIS, trainers may focus on the practice of *basic laparoscopic skills* or on acquiring *procedural skills* for a specific procedure [24] using simulators ranging from organic to nonorganic composition [13]. Employing organic simulators presents ethical and practicality issues, hence giving rise to the development of synthetic box trainers and virtual reality training systems. On the one hand, box trainers are relatively inexpensive, versatile devices and provide realistic haptic feedback. On the other hand, VR systems are easy to set up, provide instant objective feedback and give the ability to vary easily the simulated anatomy. Both categories are complementary to one another. Naturally, other innovative solutions combine box and VR technology in order to benefit from the advantages of each system [12], which are referred to as mixed-reality systems [15].

Concerning trainers for invasive fetal procedures, various attempts have been made to develop simulators with different levels of realism [3, 14, 17, 25]. However, regarding TTTS, the only existing trainer for laser therapy is a silicone simulator with proven face and construct validity [16]. Their simulator consists of a full anatomical mock-up of the abdomen of a pregnant women, replicated by high-fidelity synthetic tissue with good ultrasound properties. Originally designed for amniocentesis [17], it was modified for fetal laser surgery by inserting in the womb mock-up a monochorionic twin placenta and realistic models of twin fetuses. The simulator is then filled with water, and a silicon interface at the top of the model imitates the abdominal wall. However, the absence of sensing technology makes objective feedback based on quantitative data impossible. In addition, the fixed aspect of the placenta and womb does not offer any room for anatomical diversity.

While practice is highly important when dealing with scarce procedures, supervision and performance feedback is currently done exclusively by experts employing structured grading, e.g., GOALS, OSATS [2]. Unfortunately, experts’ time for such purposes is scarce yet extremely valuable, and as such, there is a need for systematic evaluation. One way toward objective, automatic and cost-effective skill assessment is through embedding comprehensive sensing within trainers and analyzing data captured during procedures [19]. Descriptive statistics can be computed to describe the dexterity as well as the interactions between the instrument and the environment, thus giving access to important performance aspects [22].

In this paper, we present a novel mixed-reality trainer with comprehensive sensing combining synthetic tissue mimicking the maternal abdomen and VR technology rendering the cavity inside the womb. This simulator offers the possibility to practice basic and procedural skills for TTTS laser treatment. The VR component allows for easy reconfiguration of the anatomy, while its physical aspect is close to in vivo realism. The trainer has been validated by a cohort of fetal surgeons proving its face and content validity. Finally, a use-case is presented in order to demonstrate how motion and force data captured by the simulator’s sensors provide useful information to quantitatively assess surgical skill.

## Mixed-reality simulator with comprehensive sensing

**Fig. 2:**
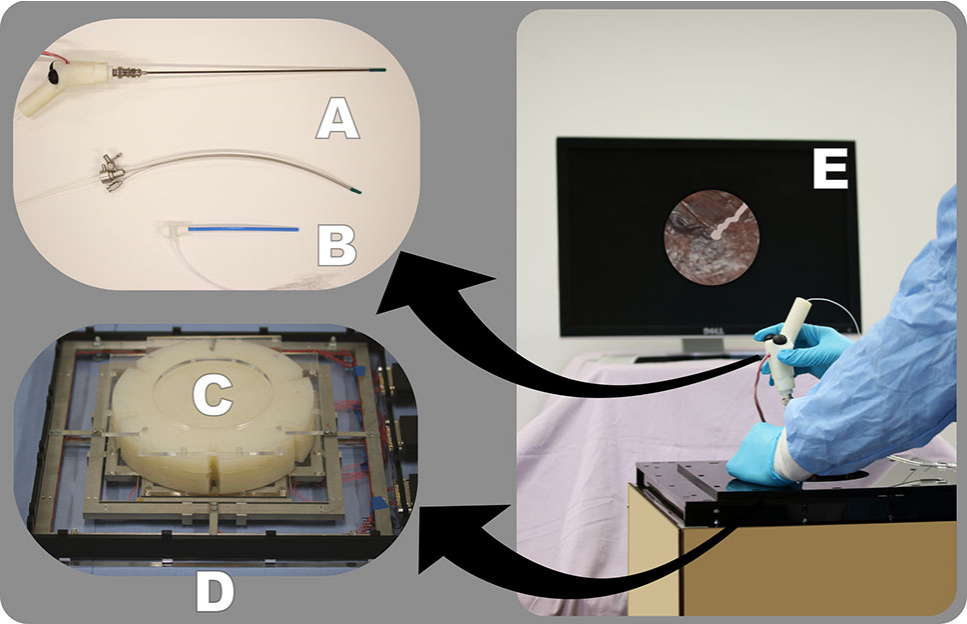
The setup of the advanced mixed-reality surgical trainer: (A) straight and curved fetoscopes, (B) cannula, (C) body wall phantom, (D) force sensor, (E) virtual scope view

For training and developing the required motor skills for TTTS surgery, we propose a novel mixed-reality surgical simulator focusing on scope handling and target lasering on the placenta. The setup consists of a fetoscope (A) which can be slid through a thin-walled flexible plastic cannula (B). The cannula is inserted in a synthetic phantom representing the maternal body wall (C) which is equipped with a force sensor (D). A cavity below the body wall allows unhindered scope motion. Contrary to a box trainer, the operator does not see on the screen images generated from the fetoscope, but instead sees artificially generated images from a virtual reality system (E). A foot pedal (not shown in the figure) is provided as well to trigger the virtual therapeutic laser. An overview of the simulator is shown in Fig. 2, and each component is further detailed below.

### Fetoscope and cannula

**Fig. 3:**
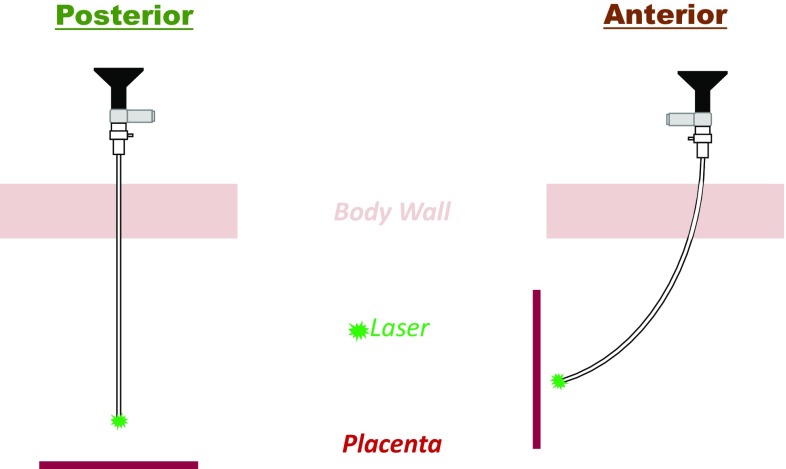
A straight fetoscope is employed for the *posterior* placenta (left). A curved one is used for the *anterior* placenta (right)

The employed surgical instrument is a fetoscope, composed of a scope inserted through a sheath. In vivo, the placenta can be located on either the *posterior* or *anterior* side of the uterus. In the former case, straight sheaths are used because a direct line of sight from the insertion point to the placenta equator exists (see Fig. 3). In the latter, no such direct line of sight exists. To solve this issue, surgeons currently employ curved sheaths bending the scope to a fixed curvature.

For this trainer, both straight and curved fetoscopes are available. As the image and laser are rendered by a VR system, it suffices to foresee small diameter beams with similar shape and compliance rather than using real scopes. Both instruments fit in a provided flexible plastic cannula with a 10 Fr diameter.

### Body wall phantom

The constraining nature of the maternal abdomen wall upon the instrument motion is an important aspect of fetal MIS. Therefore, the different layers of skin, fat, muscle, uterus and fetal membranes are replicated by a *body wall* phantom composed of several layers of synthetic material (Ecoflex 00-50, *Smooth-On Inc., USA*). The trainer allows the usage of body wall phantoms of different thickness. The cannula is inserted through a hole in its center.

### Sensors

To allow for accurate rendering in the VR system and eventually for motion analysis, instrument motions are tracked with an electromagnetic tracking system (Aurora, *NDI*, USA) composed of a field generator and a 6-degrees-of-freedom (DOF) sensor at the tip of the fetoscope. Furthermore, the interaction forces between the instrument and the body wall phantom are monitored by a dedicated 6 DOF force sensor [9]. The sensor is made of an aluminum monolithic structure and measures several deformations with strain gauges providing the measurement of 6 DOF forces which are applied on the element clamped to it, i.e., the body wall phantom. The data acquisition of the force sensor can be done through a CompactRIO Controller (*National Instruments*, USA) and a LabVIEW software (*National Instruments*, USA).

Finally, the trainer is equipped with a foot pedal whose usage is tracked over time. The role of the foot pedal is to notify the VR system of the user’s intention to activate the therapeutic laser, virtually placed at the instrument’s tip.

### Virtual reality environment

To render the fetoscopic view and immerse the user into a realistic TTTS procedure, a VR system has been developed. Currently, the emphasis of the simulator is to provide a means for practicing motor skills and, as such, the environment is slightly simplified: only the placenta and the laser ablation sites are rendered at a realistic scale in the scope view.

*The placenta* is represented as a realistically sized planar surface with a real monochorionic placenta image textured onto it. The placenta pose can easily be configured, and two default modes are already available: a placenta in a *posterior* or *anterior* pose. The VR system offers the possibility to freely edit this image by adding indicators such as arrows and symbols.

*The artificially generated scope view* is rendered on a display positioned in front of the operator at eye level, similar to a real clinical setting. The lighting attenuates with increasing distance between scope and placenta. As observed in the OR, the laser’s presence can be seen as a green dot at the center of image.

*The laser* is activated whenever the user presses the foot pedal. Lasering events apply white marks on the targeted location on the placenta surface. The distance between the instrument’s tip and the placenta, as well as the duration, determines the intensity of these marks.

The VR system is written in C++ and runs on a laptop with a Intel Core i7-4910MQ Processor and a Linux OS. The open-source Visualization Toolkit (VTK) was employed to render the graphics.^1^ In order to communicate with the required hardware, i.e., instrument tracking device and foot pedal, the Robot Operating System (ROS) was employed.

## Validation study: face and content

In this work, we aim at creating a realistic mixed-reality trainer offering a real help toward mastering TTTS required skills. As such, we perform both face and content validation studies to assess the overall trainer quality and capabilities [8]. Eight surgeons were asked to perform different tasks on the simulator followed by a two-section questionnaire.

### Questionnaire

The first section of the questionnaire gathers each participant’s background information, while the second focuses on the face and content validity. In total, 33 statements were spread into three main categories; each scored on a 5-point Likert scale. First, 14 statements evaluated the realism of each component of the trainer. For each statement, the five possible answers were: *not close, a bit close, close, very close* and *realistic*. Secondly, for each aspect of realism, the improvement required for training purposes is inquired, adding 14 statements to the questionnaire having the following five possible answers: *no improvements, minor improvements, medium improvements, major improvements* and *critical improvements*. Finally, 5 statements focused on the content validity by asking the user’s agreement based on a 5-point Likert scale ranging from *disagree* to *agree*.

### Participants

Eight surgeons were part of the study, all from the Gynaecology Department of the UZ Leuven Hospital which is the only Belgian center specialized in TTTS treatment. To put in perspective, only two exist in France, two in Germany and four in England [7].

The information gathered from the questionnaire’s first section allowed to classify participants into three different categories: *novice*, *intermediate* and *expert*. The criterion was based on their self-evaluation of their ability to either perform an operative fetoscopy independently as lead surgeon in the OR (*expert*), or perform with supervision (*intermediate*), or none of the above (*novices*). Under this classification, the groups of participants consisted of two experts, two intermediates and four novices. The experts had at least performed 300 interventions, intermediates at least 1 and the novices only assisted such surgeries. However, all of the novices had clear knowledge of the procedure, attended several fetal MIS procedures and had hands-on experience in other types of MIS.

### Tasks and protocol

Two different tasks were proposed to demonstrate the simulator’s potential for training *basic fetoscopic skills* for laser ablation in fetal surgery and *procedural skills* for TTTS surgery.

**Fig. 4:**
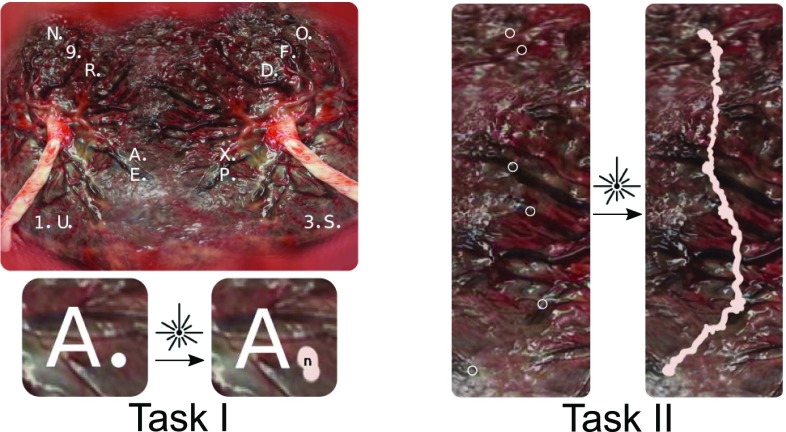
Illustration of the two different tasks with the effect of lasering on the placenta

*Task I—Basic fetoscopic skills* are designed as a series of symbols, i.e., letters and numbers, scattered over the whole placenta. The user must sequentially identify the symbols and laser the small dot placed next to each one. When the dot is completely lasered, the next symbol to target is revealed (Fig. 4).

*Task II—Procedural skills* require to perform the laser ablation of placental anastomoses using the *Solomon technique* as closely as possible to in vivo conditions [20]. Multiple anastomoses are scattered on the placenta’s equator. In order to avoid testing the participant’s cognitive skills, the anastomoses are clearly highlighted by a ring. The first part of the procedure, referred to as *selective lasering*, consists of completely lasering the inside of each ring. The second part, called the *dichorionization of the placenta*, is when the user performs a continuous lasering in between each target (Fig. 4).

To summarize, each participant performed the following steps once:

1) A 3-minute training session to familiarize with the simulator,

2) Task I with a *posterior* placenta and a *straight* fetoscope,

3) Task I with an *anterior* placenta and a *curved* fetoscope,

4) Task II with a *posterior* placenta and a *straight* fetoscope,

5) Task II with an *anterior* placenta and a *curved* fetoscope,

6) Answering the validation questionnaire.

### Statistical analysis

Considering the low number of participants and the number of groups, the nonparametric Kruskal–Wallis test was employed with a difference considered as significant when *p*-value $<0.05$. This allowed to observe whether the different groups came to the same conclusion regarding each aspect of the face and content validation. The statistical analysis was performed using MATLAB.

**Table 1:** Results of face validity

Face validity	Total	Novices		Intermediates		Experts		$p^\mathrm{a}$
	Median	Median	SD	Median	SD	Median	SD	
Overall realism of trainer	3.50	3.50	1.00	3.50	0.50	3.50	0.50	1.000
Body wall phantom	3.00	3.50	1.00	3.50	0.50	3.00	0.00	0.497
Equipment	4.00	3.50	1.00	3.50	0.50	4.00	0.00	0.497
Environment VR rendering								
Placenta model	3.00	4.00	0.25	3.00	0.00	3.00	0.00	0.123
Posterior configuration	3.50	4.00	0.25	3.00	0.00	3.50	0.50	0.269
Anterior configuration	3.50	4.00	0.25	3.00	0.00	3.00	1.00	0.343
Coagulation model	3.00	3.00	0.25	3.50	0.50	3.50	0.50	0.249
Procedural tasks								
Selective lasering	4.00	3.50	1.00	4.00	0.00	3.50	0.50	0.497
Line lasering	4.00	3.00	0.50	4.00	0.00	4.00	0.00	0.135
Scope VR rendering								
Scope view	3.00	4.00	0.25	3.00	0.00	3.00	0.00	0.123
Image quality	3.00	3.00	0.25	3.50	0.50	2.50	1.50	0.553
Light propagation	4.00	3.00	2.00	4.00	0.00	3.50	0.50	0.472
Depth perception	3.50	3.50	1.50	3.50	0.50	3.00	1.00	0.936
Workspace of instruments	3.50	3.00	0.25	4.00	0.00	3.50	0.50	0.269

${^\mathrm{a}}$Kruskal–Wallis test with significance $p<0.05$

### Results

The VR system is written in C++ and ran on a laptop with a Intel Core i7-4910MQ Processor and a Linux OS. The Robot Operating System (ROS) was used to communicate with the different hardware, i.e., the EM tracking device and the foot pedal. The data acquisition of the force sensor was done via a CompactRIO Controller (*National Instruments*, USA) and a LabVIEW software (*National Instruments*, USA). The statistical analysis was performed using MATLAB.

Results of the face validity test are represented in Table 1. Overall, surgeons considered the trainer to be close to reality, with a median score of 3.50 on the 5-point Likert scale. Most aspects of the simulator scored above 3 as well. The lowest score was given to the image quality of the scope rendering with a expert median score of 2.50. No significant difference was observed between each group as the nonparametric test gave a $p>0.05$.

**Fig. 5:**
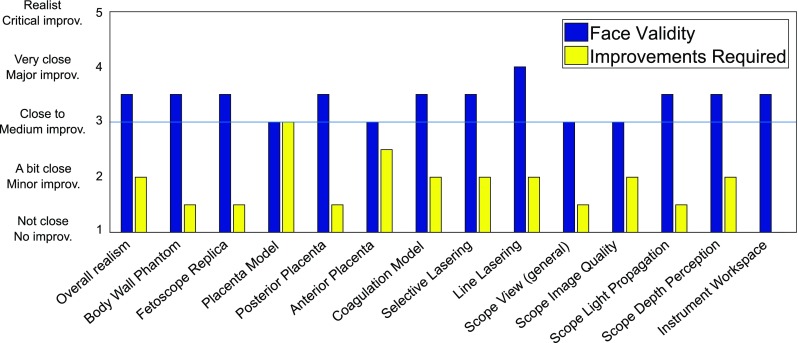
Realism versus required improvements according to fetal MIS surgeons

The comparison between the *realism* and *required improvements* can be found in Fig. 5. The placenta model requires the most attention for further developments (total improvements median: 3.00), even though it was judged with a satisfying close-to-realism score (3.00). Despite the fact that the realism of the scope’s view and its rendered image are weak, they seem to remain acceptable for training (total improvements median: 1.50 and 2.00, respectively). Finally, improvements might be interesting for the rendering of the anterior placenta as its score for improvement is of 2.50, while its realism is of only 3.00.

Regarding the content validity, results are given in Table 2. The surgeons were all convinced by the training capacities of the simulator and found the tasks relevant for improving *basic fetoscopic skills* and *procedural skills*. Indeed, the total median score of each aspect of the content evaluation was scored at the maximum value 5.00. The novices gave the maximum score for all content validity aspects, while experts lowered slightly the score. However, once again there was no significant difference between the answers of each group.

**Table 2:** Results of content validity

Content validity	Total	Novices		Intermediates		Experts		$p^\mathrm{a}$
	Median	Median	IQR	Median	IQR	Median	IQR	
Training capacities								
Scope handling	5.00	5.00	0.25	4.50	0.50	4.00	1.00	0.664
Lasering	5.00	5.00	0.00	5.00	0.00	4.50	0.50	0.223
Self-confidence	5.00	5.00	0.00	5.00	0.00	4.50	0.50	0.223
Usefulness of task								
Task I: basic skills	5.00	5.00	0.25	5.00	0.00	4.50	0.50	0.558
Task II: procedural skills	5.00	5.00	0.25	5.00	0.00	4.50	0.50	0.558

${^\mathrm{a}}$Kruskal–Wallis test with significance $p<0.05$

## Use-case: surgical hypothesis verification using comprehensive sensing analysis

The innovative aspect of the proposed trainer resides in the embedded comprehensive sensors. In order to show the potential of the simulator’s sensors to objectively assess skill, a use-case analysis has been carried out to assess a surgical hypothesis.

### Hypothesis

When treating TTTS with laser therapy, surgeons claim that operating on an *anterior* placenta increases the difficulty of the procedure compared to a *posterior* configuration. Through the questionnaire, all surgeons confirmed this statement when carrying out the different tasks on the simulator in both placenta configurations. The main difficulties pointed out during the trial were the manipulation of the curved instrument itself and accessing the different targets on the extremities of the placenta. By analyzing the different signals captured by the simulator, our objective was to see whether a similar assessment could be quantitatively observed.

### Experimental protocol

**Fig. 6:**
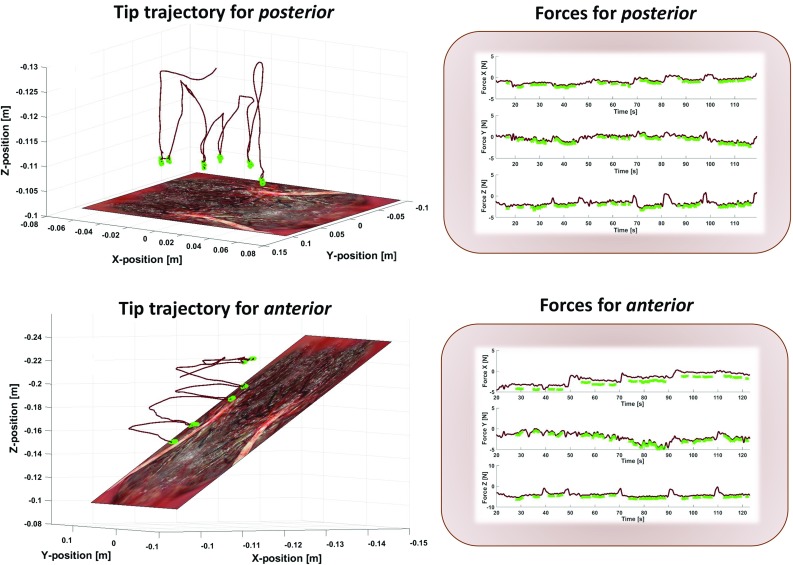
The different signals of motion, force and pedal state data are shown for selective lasering in a posterior and anterior configuration. For each plot, the signal is highlighted in green when the pedal is activated

For this study, the number of anastomoses during task II was set to six, which is a common number found *in vivo*. Each session took place with a 40-mm body wall phantom thickness as recommended by expert surgeons. During each experiment, the instrument tip motions and the interaction forces between the instrument and the body wall were both recorded. A real-time binary signal of the pedal state, i.e., *on* or *off* state, was monitored as well. In Fig. 6, all of these signals are represented (orientation data and 3 DOF moments are also available but not shown in this figure). The motion signals were filtered with an exponential smoothing, the force signals were processed with a Butterworth filter, and all signals were synchronized. Only the portions of the signal when the instrument tip was in the cavity were considered.

### Metrics

As skill assessment is not the core of this paper, commonly used motion-based [5] and force-based metrics [22] were computed. For each metric, a significance test (e.g., Kruskal–Wallis test) was conducted in MATLAB to highlight which metric evaluated a significant difference between the anterior and posterior placenta configuration.

The following set of metrics was evaluated: mean/maximum of velocity and acceleration, tool path length (length of the curve described by the tip of the instrument), depth perception (the total distance travelled by the instrument along its axis), maximum of planar/vertical force and integral of planar/vertical force (a measure of high forces and the amount of time that forces are high). *Time*, a widely used metric in skill analysis, was also computed.

**Table 3:** Results of the motion and force-based analysis of the use-case study

Metrics	$p^\mathrm{a}$	Posterior		Anterior	
		Mean	SD	Mean	SD
Planar force maximum	**0**.**031**	3.71	1.48	6.99	2.01
Planar force integral	**0**.**031**	412	125	813	153
Vertical force integral	**0**.**031**	462	387	786	375
Depth perception	**0**.**039**	1.09	0.55	1.41	0.50
Vertical force maximum	0.218	1.06	0.70	1.57	0.93
Velocity maximum	0.383	0.91	0.71	1.06	0.60
Acceleration maximum	0.641	4.20	3.77	4.42	3.04
Tool path length	0.742	2.28	1.04	2.47	0.90
Acceleration mean	0.742	0.030	0.010	0.032	0.005
Velocity mean	0.844	0.0081	0.0025	0.0084	0.0016
Time	1.000	269.62	111.53	269.38	71.30

${^\mathrm{a}}$Kruskal–Wallis test with significance bold values $p<0.05$

During those experiments, all motions were successfully captured with the exception of the force data for two surgeons. Nevertheless, these were still considered for analysis. Table 3 summarizes the results of the statistical test. The metrics tested significant ($p>0.05$) are highlighted. One may first observe that *time* brings no significant information on the increase of difficulty between the anterior and posterior configurations.

However, force-based metrics appear to be significantly different between the anterior and posterior configurations. The maximum planar forces and their integral double in average. The same can almost be seen for the integral of the vertical forces. Finally, with an increase of almost 30%, depth perception seems to be the only motion metric to identify a significant increase of difficulty.

## Discussion

In this study, the trainer was validated by using face and content validity as a first step. Even though these tests of validity are a subjective approach to validation, it was enough to confirm the simulator’s overall closeness to reality in all the main aspects as well as improvement opportunities. Most surgeons indicated that training with the current status of the simulator may already facilitate the learning of the required skills. Objective approaches for evaluation also exist, such as construct validity which aims to determine whether a simulator can discriminate between different levels of expertise [21]. This next step of validation was left aside for now as we believe that surgeon support and feedback is important before advancing to experiments objectively assessing the simulator. Upon further development of the placenta rendering (i.e., availability of various placentas with different morphology inducing an increase in difficulty) and the visualization (i.e., turbidity of the amniotic fluid), we believe performing a construct validity will yield more powerful results.

The data available are very scarce because only specialized centers currently treat such pathology. The group size in this study is rather small in order to perform statistical correlations. However, we do believe it is enough to gain insights on the performance of the trainer and how it should be improved. This trainer should preferably be investigated further by other centers specialized in TTTS treatment in order to enlarge the sample of the study and confirm the mentioned conclusions.

Surgeon feedback is crucial for the development and assessment of simulators. Even though it has a subjective aspect, it is possible to extract conclusions by inquiring the correct group of people through the means of a well-built questionnaire. As identified by Schout et al., the literature shows a lack of consensus on the correct approach to be followed. We believe the choice of a five-point Likert scale was adequate for this study, and it seemed well suited to inquire a specialized center in TTTS with surgeons of different levels of expertise.

Regardless of the small group size, we decided to demonstrate the simulator’s potential to objectively evaluate performance through a use-case study. A largely used metric for assessment is *time*. For this dataset, this metric was unable to measure anything significant, while motion and force-based metrics revealed to be crucial in order to obtain an objective assessment on surgical skill. However, not all computed metrics passed the significance test. It is important to state that our low number of total trials makes it difficult to measure significantly a difference between anterior and posterior configurations.

## Conclusion and future work

A novel mixed-reality surgical trainer with comprehensive sensing for fetal MIS was presented in this paper. Validated by a group of surgeons from a specialized center in fetal medicine, the system is perceived as a good means for practicing basic fetoscopic skills and procedural skills for laser surgery. Further similar investigations in other specialized centers are of interest, as well as quality improvements. Ensuing proper adjustments, we believe the system capable to be used as a platform to conduct motion and force-based skill analysis.

## Footnotes

1 For more information on the software, feel free to contact the corresponding author.
